# Membrane Vesicles as Drug Delivery Systems: Source, Preparation, Modification, Drug Loading, In Vivo Administration and Biodistribution, and Application in Various Diseases

**DOI:** 10.3390/pharmaceutics15071903

**Published:** 2023-07-07

**Authors:** Chenhan Sun, Ying Qin, Hongda Zhuang, Yuan Zhang, Zhiwen Wu, Yong Chen

**Affiliations:** Jiangxi Key Laboratory for Microscale Interdisciplinary Study, Institute for Advanced Study, Nanchang University, Nanchang 330031, China; sunchenhan0708@126.com (C.S.); qinying1013@hotmail.com (Y.Q.); hongdadazhuang@gmail.com (H.Z.); 15279115010@163.com (Y.Z.); 15179658052@163.com (Z.W.)

**Keywords:** drug delivery systems (DDSs), membrane vesicles, extracellular vesicles (EVs), exosomes, bacterial outer membrane vesicles (OMVs)

## Abstract

Bioinspired (or biologically inspired) drug delivery systems (DDSs) have been intensively studied in the last decades. As bioinspired DDSs, membrane vesicles, including extracellular vesicles (EVs) released from eukaryotic cells, outer membrane vesicles (OMVs) from bacteria, cell-bound membrane vesicles (CBMVs) isolated in situ from cell surfaces, membrane vesicles reorganized after the isolation of the plasma membrane of cells, and others have been rapidly developed and are attracting more and more attention. Most recently, a collection of 25 papers on the advances in membrane vesicle-based drug delivery systems was published in a Special Issue of *Pharmaceutics* entitled “Advances of membrane vesicles in drug delivery systems”. These papers cover many related topics including the source, preparation, modification, drug loading, and in vivo administration and biodistribution of membrane vesicles (mainly extracellular vesicles or exosomes and bacterial outer membrane vesicles), as well as application of membrane vesicles as DDSs in the treatment of various diseases.

## 1. Introduction

**Figure 1:**
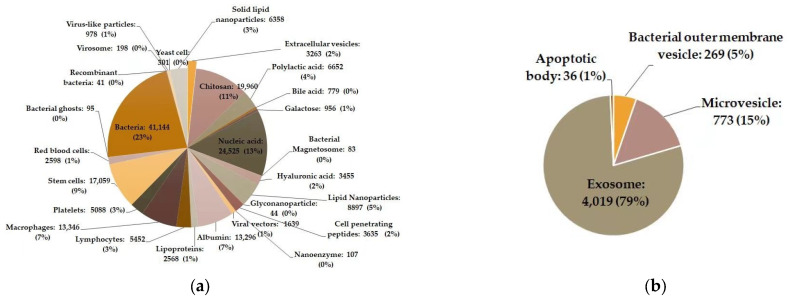
Publications of different types of bioinspired drug delivery systems (DDSs)**.** (**a**) 27 types of bioinspired DDSs; (**b**) Main types of membrane vesicles as DDSs including extracellular vesicles (EVs which are divided into three subtypes: Exosome, microvesicle or microparticle, and apoptotic body) and bacterial outer membrane vesicles. The data were obtained from the Web of Science database by searching for article title or the abstract field using the following keywords: ‘Drug delivery’ or ‘drug carrier’ and ‘the name of a drug delivery system’ (publication data is as of 18 June 2023).

Drug delivery systems (DDSs) are defined as formulations or devices that deliver therapeutic substances specifically to their sites of action without reaching nontarget sites (cells, tissues, or even organs). Many benefits to drugs can be achieved by drug delivery systems, including an elevated stability, higher water solubility, prolonged circulation time, better sustained drug release, stronger tissue/cell targetability, lower drug dosage, impaired side effects, enhanced efficacy, etc. Over the latest two decades, bioinspired (or biologically inspired) drug delivery systems have been intensively studied. Compared with traditional DDSs, the advantages of bioinspired DDSs include lower/no immunogenicity, biocompatibility, biodegradability, better safety, among others. By mimicking (or being prepared from) natural components inside the body, many bioinspired DDSs have been tested, including biomolecule (e.g., peptides)-, lipid-protein complex (e.g., reconstituted high-density lipoprotein [1,2]), membrane vesicles-, virus-, and even whole cell (e.g., bacterium, red blood cell, platelet, stem cell)-inspired DDSs (Figure 1a shows 27 types of bioinspired DDSs frequently studied in recent decades).

**Figure 2:**
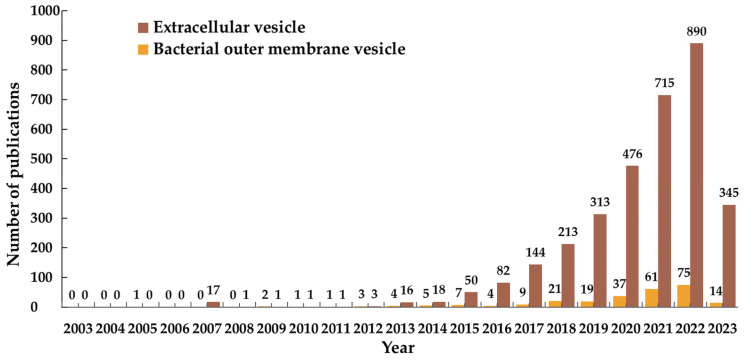
Publications by year of extracellular vesicles (EVs) and bacterial outer membrane vesicles (OMVs). The data were obtained from the Web of Science database by searching for article title or the abstract field using the following keywords: ‘Drug delivery’ or ‘drug carrier’ and ‘the name of a drug delivery system’ (the timespan is from 1 January 2003 to 18 June 2023).

As one specific type of bioinspired DDSs, membrane vesicles have a membranous structure derived from natural cells. Several types of membrane vesicles have been reported including well-known extracellular vesicles (EVs) released from eukaryotic cells, outer membrane vesicles (OMVs) from bacteria, cell-bound membrane vesicles (CBMVs) isolated in situ from cell surfaces [3,4,5], membrane vesicles reorganized after the isolation of the plasma membrane of cells, etc. Extracellular vesicles are generally divided into three categories: exosomes, microvesicles or microparticles, and apoptotic bodies among which the research on exosomes predominates in the research field of extracellular vesicles (Figure 1b). The bearing of some specific molecules, which originally exists on/in the source cells enables membrane vesicles to have specific functions (e.g., making the target cells have some properties of the source cells). In recent decade, membrane vesicles, particularly extracellular vesicles and bacterial outer membrane vesicles, have been rapidly developed as inspired DDSs (Figure 2 displays the EV-/OMV-related publications by year). In the Special Issue “Advances of membrane vesicles in drug delivery systems” of the Journal *Pharmaceutics* which belongs to the section “Drug Delivery and Controlled Release”, 25 papers on the recent advances in membrane vesicle-based drug delivery systems have been collected and arranged in this Editorial article in the following order: the source, preparation, modification, drug loading, and in vivo administration and biodistribution of membrane vesicles (mainly extracellular vesicles or exosomes and bacterial outer membrane vesicles), as well as application of membrane vesicles as DDSs in the treatment of various diseases.

## 2. Sources of Membrane Vesicles for DDSs

The selection of the sources for membrane vesicle production is crucial and needs to be prioritized during studies since it determines the composition and biological activity of membrane vesicles and influences the functional state of membrane vesicles as DDSs. Some studies have shown that extracellular vesicles exerted a better therapeutic efficacy than the cells releasing the EVs [6]. At present, abundant sources of membrane vesicles have been developed, including various biological fluids (e.g., blood, milk [7,8], orange juice [9]), animal cells (e.g., mesenchymal stem/stromal cells (MSCs) [7,10,11], dendritic cells [12], macrophages [13], tumor cells [14], red blood cells [15], different animal cell lines [16], and others), plant cells (e.g., grapes, conifers, ginger, garlic, tea, etc.) [17], bacteria (e.g., intestinal flora and probiotics) [18,19,20], and others [21,22]. Among the abovementioned sources, MSCs are widely used because of their pluripotency and the ability of differentiation into a variety of cell types; cell sources, e.g., γδ T cells and macrophages, are also the best sources for EVs targeting cancer cells [23]; Recently, researchers are paying more attention to developing new natural membrane vesicle sources and engineered EVs [16]. 

## 3. Preparation and Modification of Membrane Vesicles as DDSs

The preparation of membrane vesicles is important for the following characterizations and applications of membrane vesicles as DDSs, and high vesicle yield and separation efficiency are required for practical production of membrane vesicles. The methods for membrane vesicle preparation should have the characteristics of robustness, ease of use, economy, repeatability, time-saving, and high throughput, and sometimes depend on the sources of membrane vesicles to a certain degree. The frequently used methods include differential ultrafiltration centrifugation (dUC), density gradient centrifugation, ultrafiltration (UF), precipitation, dimensional exclusion chromatography (SEC), and immunoaffinity capture, as well as emerging technologies (e.g., microfluidic) [6]. In this Special Issue, commercial ExoQuick-Tc^TM^ Exosome Precipitation Solution was used for the isolation of dendritic cell-derived exosomes [12] and of human cell-secreted EVs [16]; differential ultracentrifugation method was used to separate EVs from mesenchymal/stromal stem cells [7] or from orange (*Citrus sinensis*) juice [9]; and density gradient centrifugation method was also utilized to prepare milk EVs [7]. 

Physiochemical or biological modification can improve the targeting, bioactivity, kinetics, and biodistribution of membrane vesicles. At present, multiple methods have been applied for membrane vesicle modification, including modification of donor cells (e.g., genetic engineering modification), direct modification of isolated vesicles (e.g., chemical modification), combination strategy, and others [10,20]. In this Special Issue, modification of donor cells by regulating the expressions of CP05 [24], TRAIL [23], CD81 [16], CCR7 [12], etc., direct modifications of isolated EVs by using iRGD, hyaluronic acid, and folic acid [8], and combination modifications with αvβ3 integrin subunit and iRGD [23] were applied. Akbari et al. reviewed the genetic and chemical functionalization of exosomes for tumor-targeted drug delivery [25].

## 4. Drug Loading of Membrane Vesicles as DDSs

It has been proved that membrane vesicles of various origins contain specific biomolecules. For example, extracellular vesicles can carry proteins, lipids, nucleic acids, and other signaling molecules to neighboring and distant cells, affecting various physiological and pathological functions of the recipient and parental cells, and participating in the development of various diseases. Not only can membrane vesicles be used as a “messenger” to modulate cells, but also as a “shooter” to target diseases. As a promising tool for drug delivery, membrane vesicles have been recruited to load different types of drugs. In this Special Issue, curcumin was loaded in extracellular vesicles for neuroinflammation [13]; peroxidase and dopamine for Parkinson’s disease [26]; lncRNA H19 for osteoporosis [14]; miRNA-210 and miRNA-132 for cardiac repair [10]; paclitaxel for head and neck squamous cell carcinoma [23]; and even CRISPR/Cas9 for gene editing [27]. Similarly, different drugs were loaded by bacterial membrane vesicles as DDSs for disease treatment. For example, CuS and levofloxacin were loaded into OMV secreted by *E. coli* and *A. baumannii*, respectively [18]. Moreover, the potential of membrane vesicles derived from probiotics as DDSs is reviewed by Krzyzek et al. [19].

## 5. Characterization of Membrane Vesicles as DDSs

With the rapid development of membrane vesicles, corresponding characterization methods have been developed. The characterization methods can be utilized according to the physical properties of membrane vesicles including vesicle structure, size, buoyancy density, optical property, zeta potential, among others [28]. A survey shows that western blotting, single particle tracking, and electron microscopy are the three most widely used techniques for the characterization of extracellular vesicles [29]. The current guidance recommendations on extracellular vesicles or Minimal Information for Studies of Extracellular Vesicles (“MISEV”) have been proposed first in 2014 (“MISEV2014”) and updated in 2018 (“MISEV2018”) by the International Society for Extracellular Vesicles (ISEV) [30] and widely recognized. According to the guidance recommendations, characterization of extracellular vesicles is mainly divided into three categories: Quantification, General characterization, and Characterization of individual extracellular vesicles based on which various techniques are required (Table 1 [30]). There is also the MISEV revision and update of the MISEV2018 in 2022, and the newest recommendations are expected to be released in 2023, whose draft (“MISEV2022 Draft”) has been shown to the public. Compared to “MISEV2018”, it is added in the characterization aspect of the draft that researchers should provide their assay’s limit of detection when EVs are being characterized with quantitative metrics. In this Special Issue, Tolomeo et al. used tunable resistive pulse sensing (TRPS) to measure EV particle size [11]; Zhang et al. utilized single particle interferometric reflectance imaging sensing (SP-IRIS) technique for capturing EVs and recording their particle size [16].

## 6. In Vivo Administration and Biodistribution of Membrane Vesicles as DDSs

Studying the in vivo drug administration and biodistribution of DDSs has a constructive role for membrane vesicles. In this Special Issue, Tolomeo et al. explored the biodistribution patterns of membrane vesicles derived from human mesenchymal stromal cells (MSCs) following different administration routes, including intratracheal, intranasal, and intravenous injections [11]. Using fluorescently labeled MSC-derived EVs in a mouse model, they revealed that regardless of the administration route, EVs exhibit widespread distribution in various organs such as the lungs, spleen, liver, and kidneys. However, the biodistribution patterns differ among the administration routes. The findings will contribute to our understanding of the administration strategies and biodistribution of membrane vesicles as DDSs, paving the way for further optimization and clinical translation of these promising drug carriers.

## 7. Applications of Membrane Vesicles as DDSs for Treating Different Diseases

**Figure 3:**
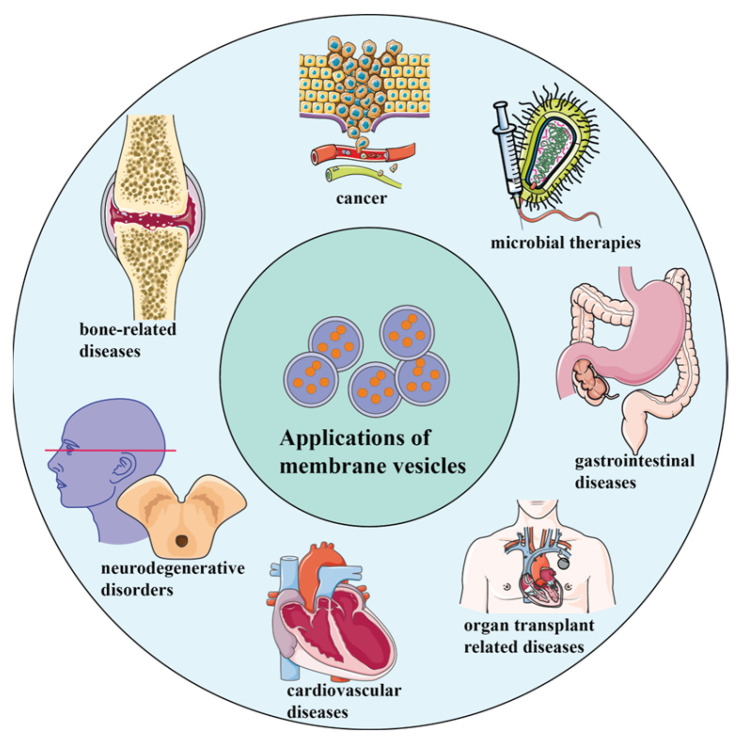
Membrane vesicles as DDSs in the treatment of different diseases.

With the development of different types of membrane vesicles, almost all types of membrane vesicles were tested to serve as DDSs or drug carriers for treating various diseases, shedding light on the therapeutic potential of membrane vesicle-based drug delivery systems across various medical conditions. A lot of related studies have been reported and summarized in many review articles. In this Special Issue, the applications of membrane vesicles as DDSs in bone-related diseases [14], anticancer and antimicrobial therapies [15,18,21,25,27], gastrointestinal diseases [17,19], organ transplant related diseases [31], cardiovascular diseases [10,12,32], neurodegenerative disorders [13,26,33], neurological anomalies associated with multidrug-resistant superbugs [20], and others were reviewed (Figure 3). Since the application of probiotics in myocardial infarction has been reported [34], it seems also potential that the membrane vesicles derived from probiotics can be recruited as DDSs to treat cardiovascular diseases (e.g., myocardial infarction). The potential impact of membrane vesicle-based therapies in these diseases is significant, as demonstrated by the studies reviewed.

Membrane vesicles (particularly extracellular vesicles and bacterial membrane vesicles) serve as effective carriers for delivering specific cell-derived molecules or therapeutic molecules or drugs, leveraging their targeted nature and intercellular communication properties [9,11,25]. The advances in recent years including those in this Special Issue will pave the way for the clinical translation of membrane vesicle-based drug delivery systems, promising improved treatment outcomes for patients. However, further studies are necessary to refine membrane vesicle production and purification methods, optimal drug loading approaches, more advanced characterization techniques, and in-depth applications in more diseases.

## Figures and Tables

**Table 1:** A brief summary of the characterization recommendations by the International Society for Extracellular Vesicles (ISEV) which is extracted from “MISEV2014” and “MISEV2018” (see the reference [30] for detailed information).

Category	MISEV2014	MISEV2018
**Quantification**	No recommendations	**Both the source of EV and the preparation of EV must be described quantitatively.** **Sourse:** Number of cultured cells, total starting volume of biofluid, or weight/volume/size of tissue. **Preparation:** Total protein amount, total particle number, total lipid quantification and the ratios among these.
**General characterization**	Analysis of the protein composition of EVs requires “three positives and one negative”, with at least three positive protein markers containing at least one transmembrane/lipid bound protein, and one cytoplasmic protein; at least one negative protein marker.	Still valid but the categories of EV proteins to characterize have evolved. Mainly used for property identification, assessment of EV purity, subtype differentiation.
**Characterization of individual EV**	Use of 2 different but complementary techniques, such as electron microscopy and single particle analysis instruments (not electron microscope-based)	Still valid but techniques used to analyze EVs have envolved.
**Additional characterization**	---	The topology of EV associated components should be assessed

## Data Availability

All data generated or analyzed are included in this article.
